# Supplementary material for: Multi-batch single-cell comparative atlas construction by deep learning disentanglement
Source: Nat Commun. 2023 Jul 12;14:4126. doi: 10.1038/s41467-023-39494-2 (PMC10336139; doi:10.1038/s41467-023-39494-2)
Supplement: Supplementary file 1 — Supplementary Information [file 41467_2023_39494_MOESM1_ESM.pdf]

Multi-batch Single Cell Comparative Atlas Construction by Deep Learning Disentanglement

Allen W. Lynch[1,2], Myles Brown[3,4] and Clifford A. Meyer[*2,5]

[1] Department of Biomedical Informatics, Harvard Medical School, Boston MA, USA
[2] Department of Data Science, Dana-Farber Cancer Institute, Boston MA, USA.
[3] Center for Functional Cancer Epigenetics, Dana-Farber Cancer Institute, Boston, MA, USA.
[4] Department of Medical Oncology, Dana-Farber Cancer Institute, Brigham and Women's Hospital, and Harvard Medical School, Boston, MA, USA.
[5] Department of Biostatistics, Harvard T.H. Chan School of Public Health, Boston, MA, USA.

[*]Correspondence to: cliff_meyer@ds.dfci.harvard.edu

This file includes:
Supplementary Table 1
Supplementary Figures 1-10

Dataset	Data type	Number of cells	Number of batches	Covariates modeled
NEURIPS bone marrow	Pure multimodal scRNA-seq + scATAC-seq	60,000	13	Donor, sequencing center, FRIP score (ATAC only)
Frankencell	Simulated scRNA-seq	4,000	2	Batch only
Embryo differentiation	scRNA-seq	150,000	6	Batch, “Is wildtype”, “Is chimera”, “Is mixed embryos” - one batch was created by mixing together cells from multiple embryos.

Supplementary Table 1. Attributes of datasets used in CODAL benchmarking experiments

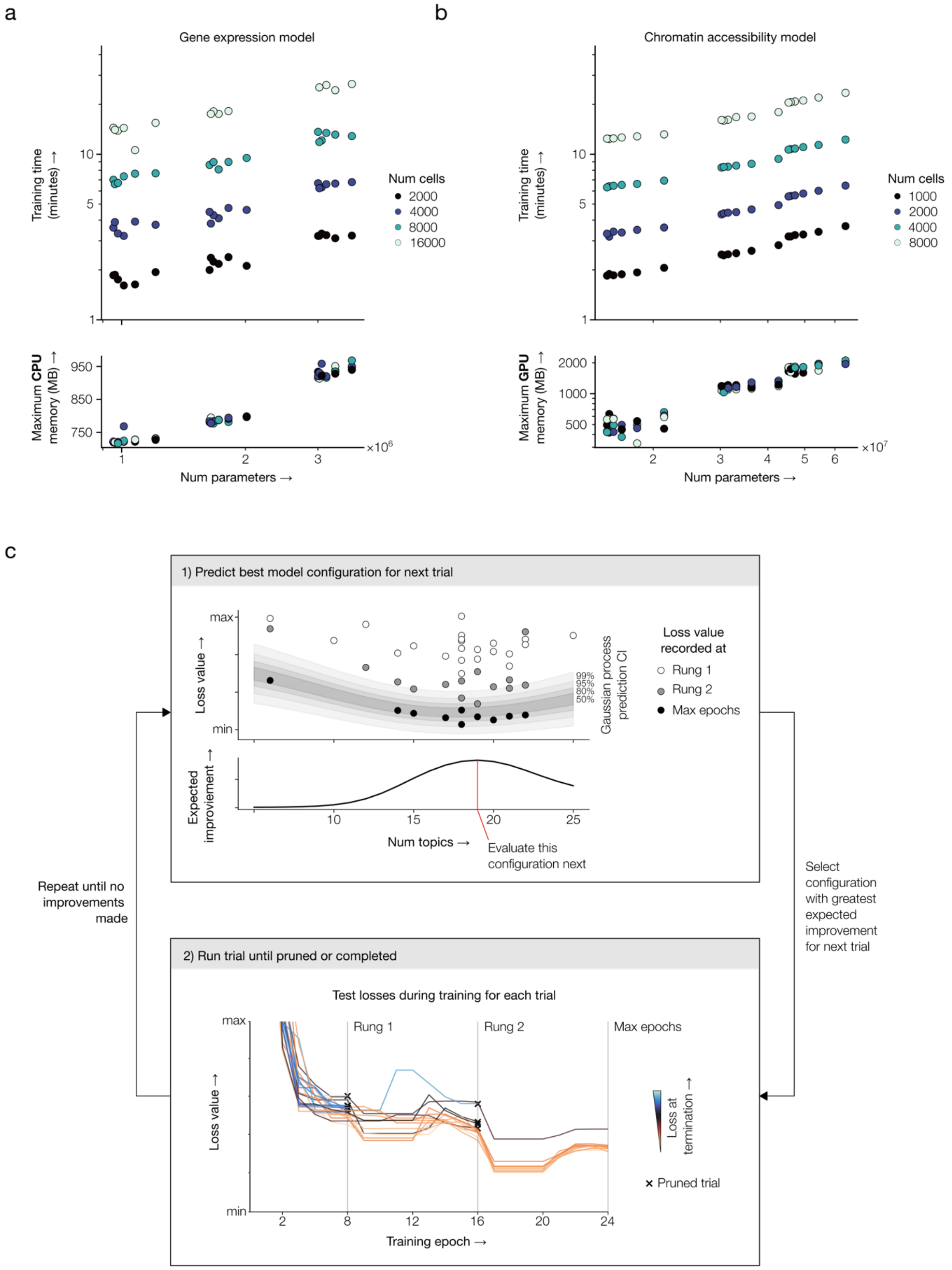

Supplementary Figure 1. Benchmarking computational resources usage and hyperparameter optimization overview

a) Computational resources required to train CODAL gene expression topic model. (top) Time to train a single CODAL model on datasets composed of two equally sized batches with varying total number of cells. We trained a model for each combination of dataset size, number of topics (4, 8, 16, 32, 64, 128), and number of

features (1000, 2000, 4000). (bottom) Maximum CPU memory usage during training for each model. **b)** Computational resources required to train CODAL chromatin accessibility model on RTX 2070 Super GPU for each combination of dataset size, number of topics, and number of features (50K, 100K, 150K). (bottom) Maximum GPU memory usage during training for each model. **c)** Overview of the iterative Bayesian hyperparameter optimization scheme for the CODAL model. (top) At each iteration, a gaussian process model is fit to the loss values recorded at each rung for all previously completed trials. Using the gaussian process approximation of the objective function, CODAL evaluates the expected improvement acquisition function for 300 randomly generated hyperparameter configurations. The configuration with the greatest expected improvement is selected for the next trial. (bottom) The model with the best predicted configuration is instantiated and trained on the "train" partition of the dataset. After each of 24 epochs, the model is evaluated using a held-out "test" partition. At epochs 8 and 16, if the model does not score in the best 50% of models trained so far, the trial is "pruned", or training is discontinued. This process is repeated until no trials improve on the best score for a set number of iterations, or the maximum number of iterations is reached. Source data are provided as a Source Data file.

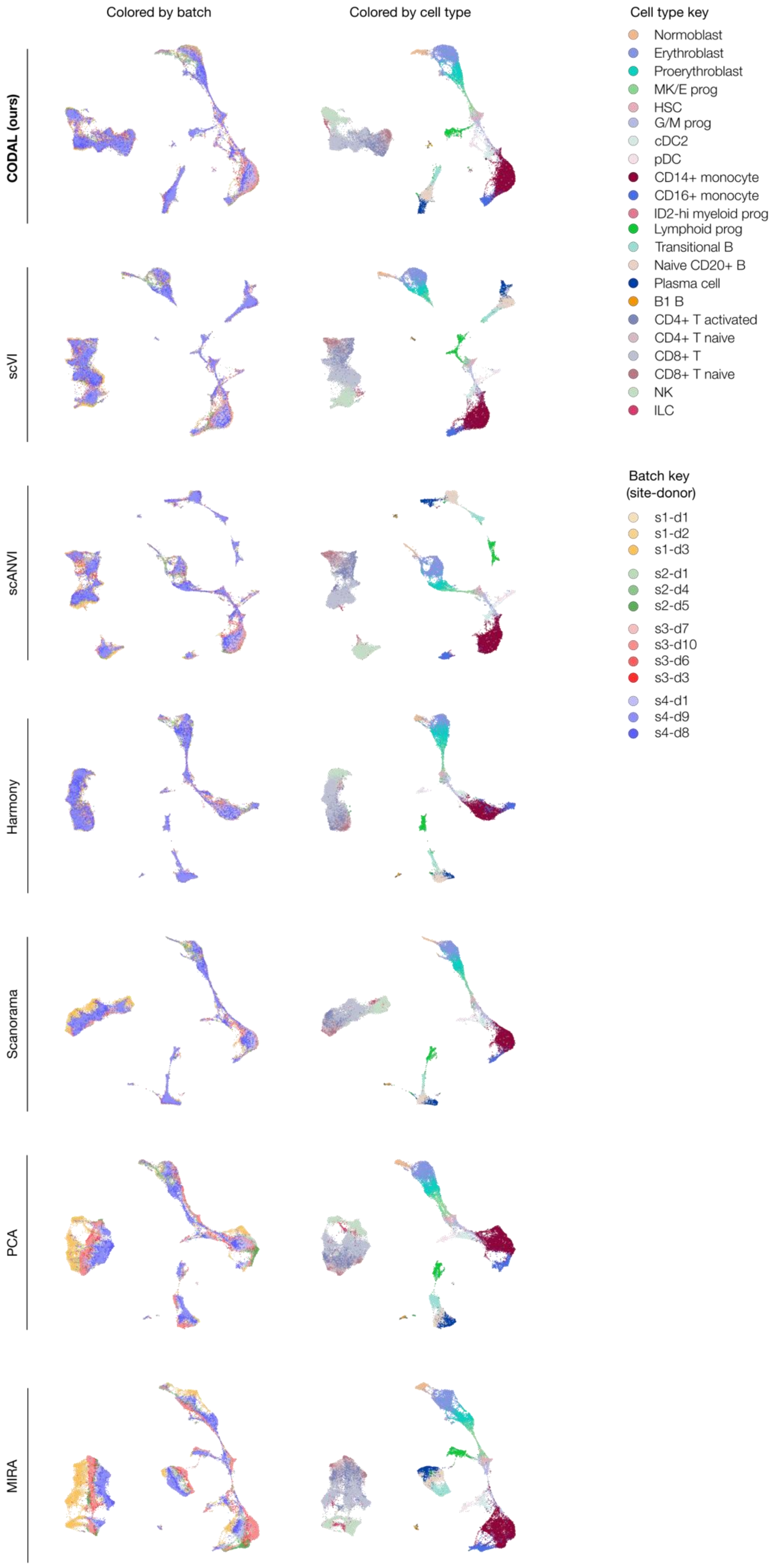

Supplementary Figure 2. UMAP representations of NEURIPS bone marrow gene expression using all methods

(left) UMAPs of latent spaces calculated from NEURIPS bone marrow gene expression dataset using all methods benchmarked, colored by batch. (right) UMAPs colored by expert-annotated cell types. Source data are provided as a Source Data file.

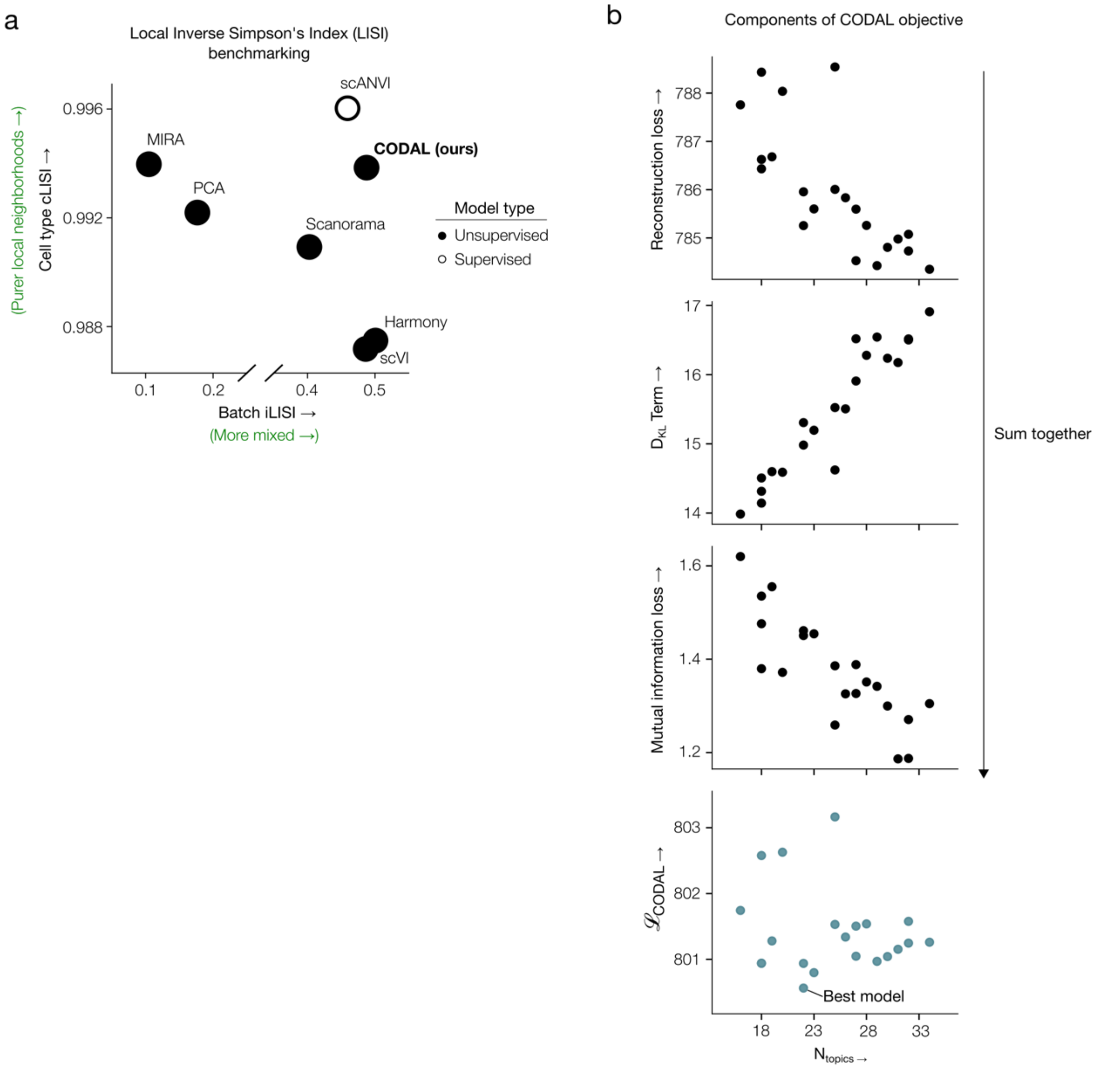

Supplementary Figure 3. NEURIPS bone marrow gene expression LISI benchmarking and model tuning

a) Benchmarking NEURIPS bone marrow gene expression latent spaces using Local Inverse Simpson's Index (LISI). Increasing cell type LISI (cILSI) corresponds with greater purity of local latent space neighborhoods based on annotated cell types. Increasing batch/integration LISI (iLISI) corresponds with greater diversity of batches in cells' local latent space neighborhoods. **b)** Additive components of CODAL loss function (the negative CODAL objective function) for models trained while tuning hyperparameters for the gene expression bone marrow dataset. Reconstruction loss is the negative log-probability of generating real data conditioned on the latent variable estimates from the encoder model. Reconstruction loss decreases with increasing N_{topics}, as greater latent space dimensionality confers increased representational capacity. The D_{KL} Term is the Kullback-Leibler divergence between the distribution of the latent variables, *Z*, and the prior for *Z*. This serves as a penalty on the amount of information encoded in the latent space since encoding information drives *Z* further from its prior, and increases with N_{topics}. Mutual information loss is the estimated mutual information between the expression rate and technical effect predictions. These components sum together to produce the CODAL loss function, which possesses a minimum with respect to the number of topics allocated to a model. This enables hyperparameter tuning to find the optimal number of topics to represent a dataset. Source data are provided as a Source Data file.

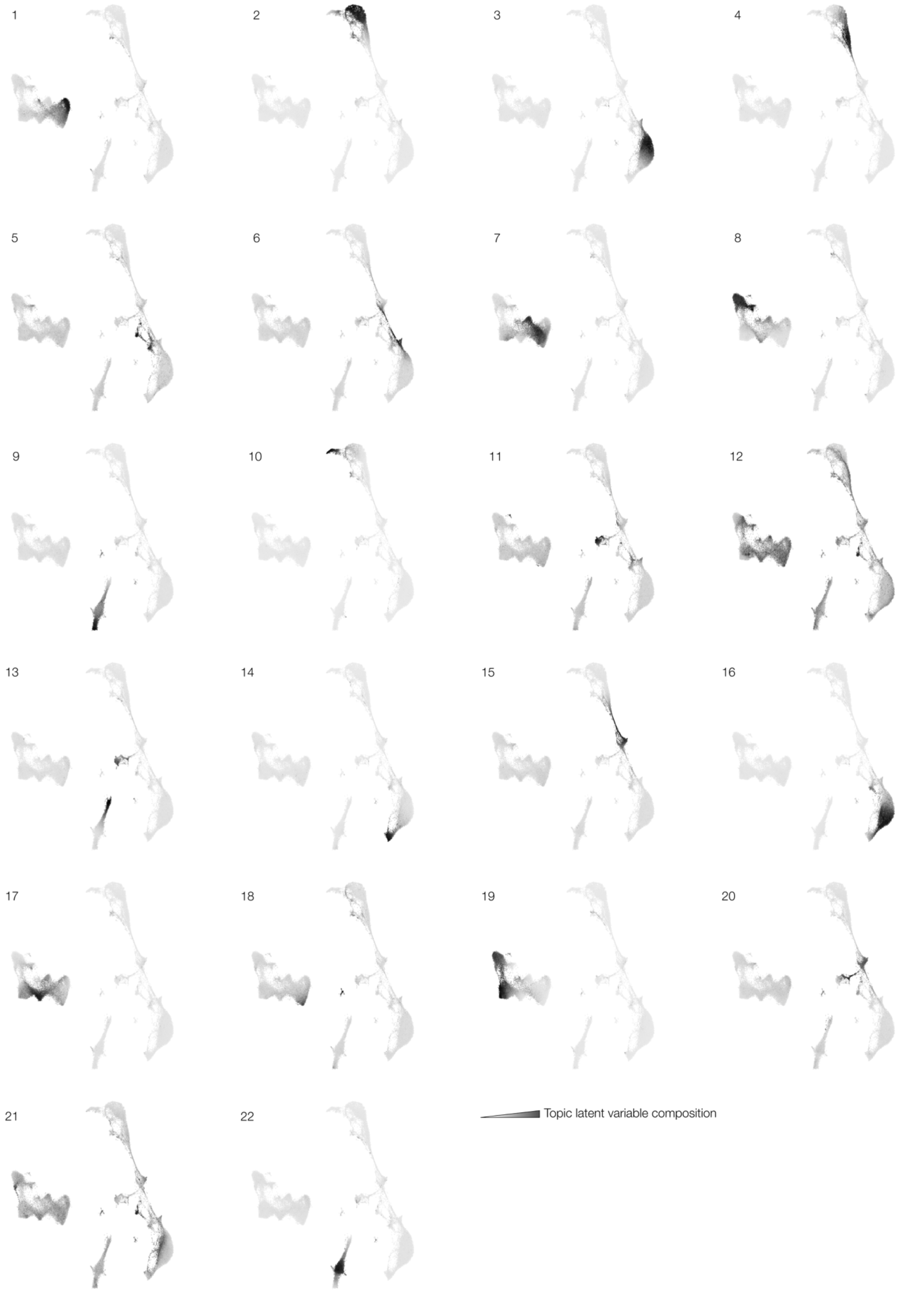

Supplementary Figure 4. CODAL latent topics from NEURIPS bone marrow gene expression

UMAPs colored by all (n=22) latent topics, *Z*, calculated from NEURIPS bone marrow gene expression dataset using CODAL. Source data are provided as a Source Data file.

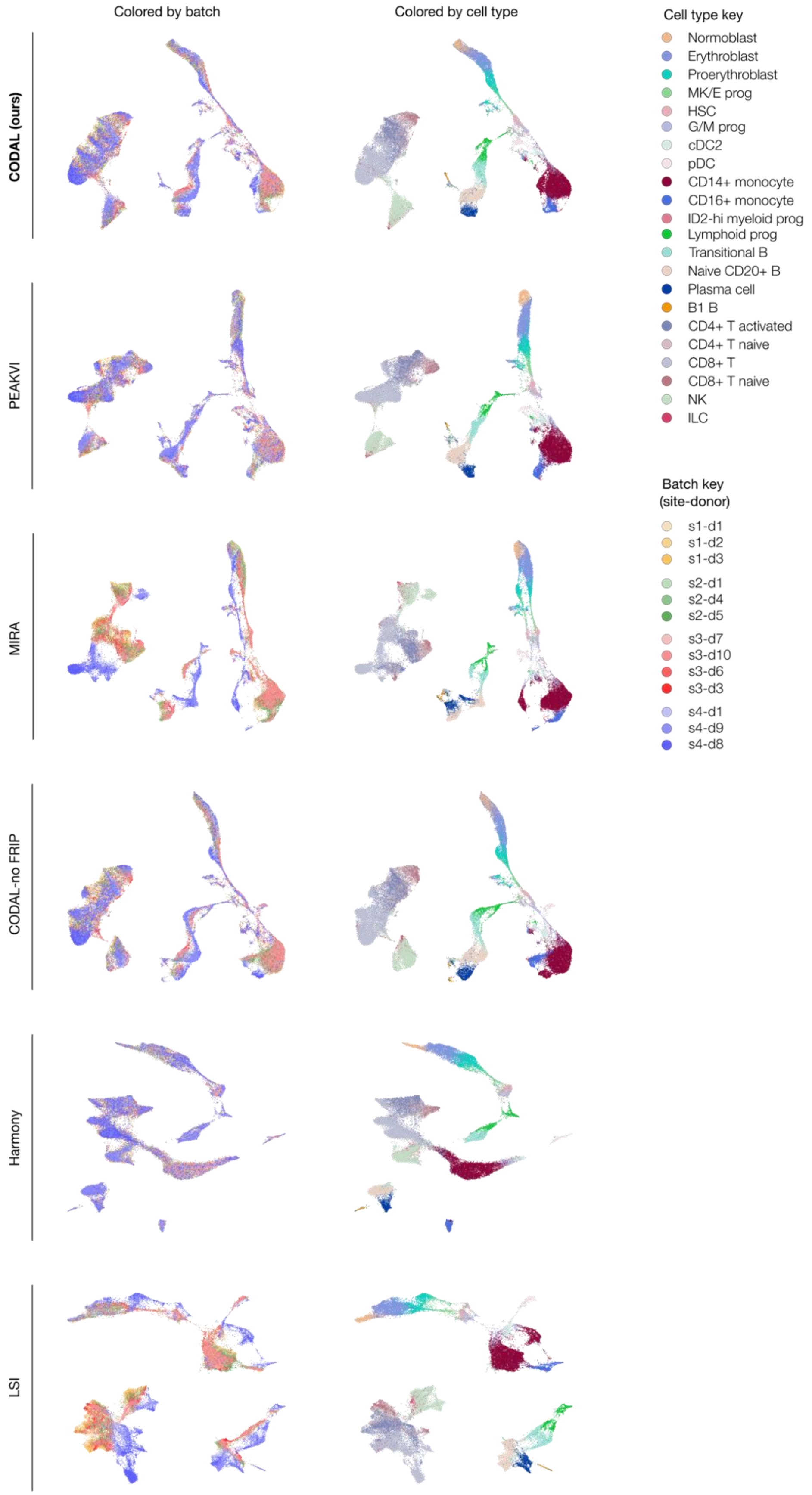

Supplementary Figure 5. UMAP representations of NEUIRIPS bone marrow ATAC using all methods

(left) UMAPs of latent spaces calculated from NEURIPS bone marrow ATAC dataset using all methods benchmarked, colored by batch. (right) UMAPs colored by expert-annotated cell types. Source data are provided as a Source Data file.

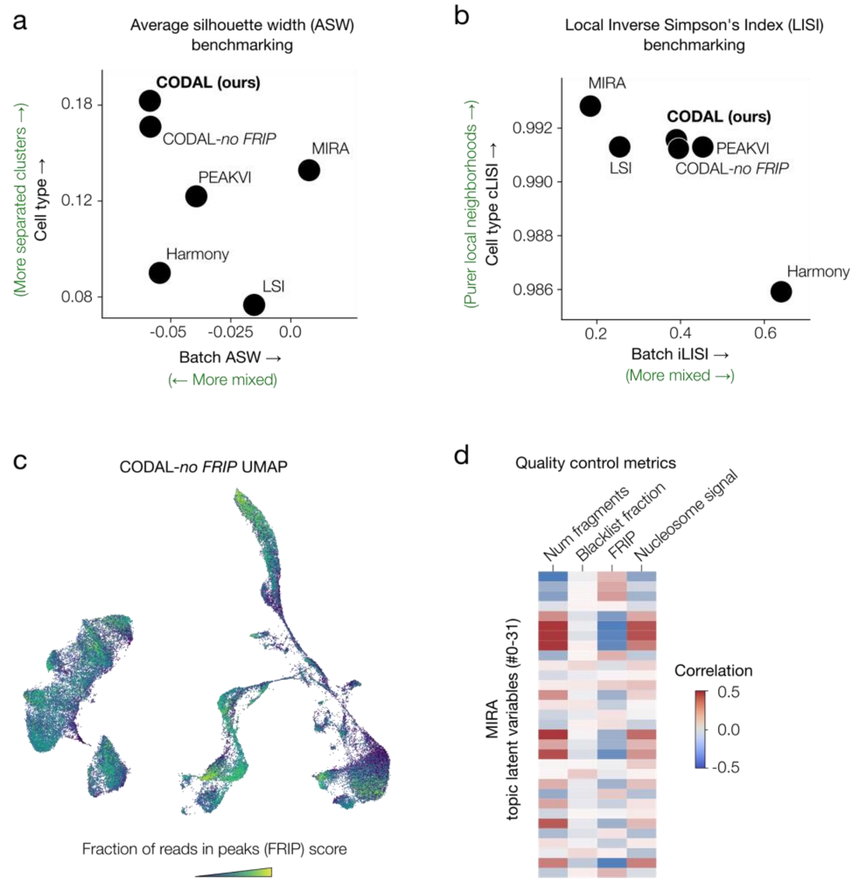

Supplementary Figure 6. NEURIPS bone marrow ATAC benchmarking and FRIP confounding analysis

a) Benchmarking cell type and batch average silhouette widths (ASW) calculated from NEURIPS bone marrow ATAC latent spaces using multiple methods. Increasing cell type ASWs correspond to greater similarity between cells with the same annotation. Decreasing batch ASWs corresponds to more mixing of cells from different batches. MIRA is a baseline topic modeling algorithm which does not correct for technical effects. CODAL-*no FRIP* corrected for batch of origin only and did not include fraction of reads in peaks (FRIP) as a technical covariate. LSI is Latent Semantic Indexing, a singular value decomposition-based method which does not correct for technical effects. **b)** Benchmarking NEURIPS bone marrow ATAC latent spaces using Local Inverse Simpson's Index (LISI). Increasing cell type LISI (cILSI) corresponds with greater purity of local latent space neighborhoods based on annotated cell types. Increasing batch/integration LISI (iLISI) corresponds with greater diversity of batches in cells' local latent space neighborhoods. **c)** Fraction of reads in peaks (FRIP) score per cell, shown on UMAP of latent space calculated by CODAL model without correcting for FRIP as a covariate. Cell type populations show different levels of FRIP score. **d)** Pearson's correlation between topics from MIRA model trained without technical effect correction versus commonly used quality control metrics. Source data are provided as a Source Data file.

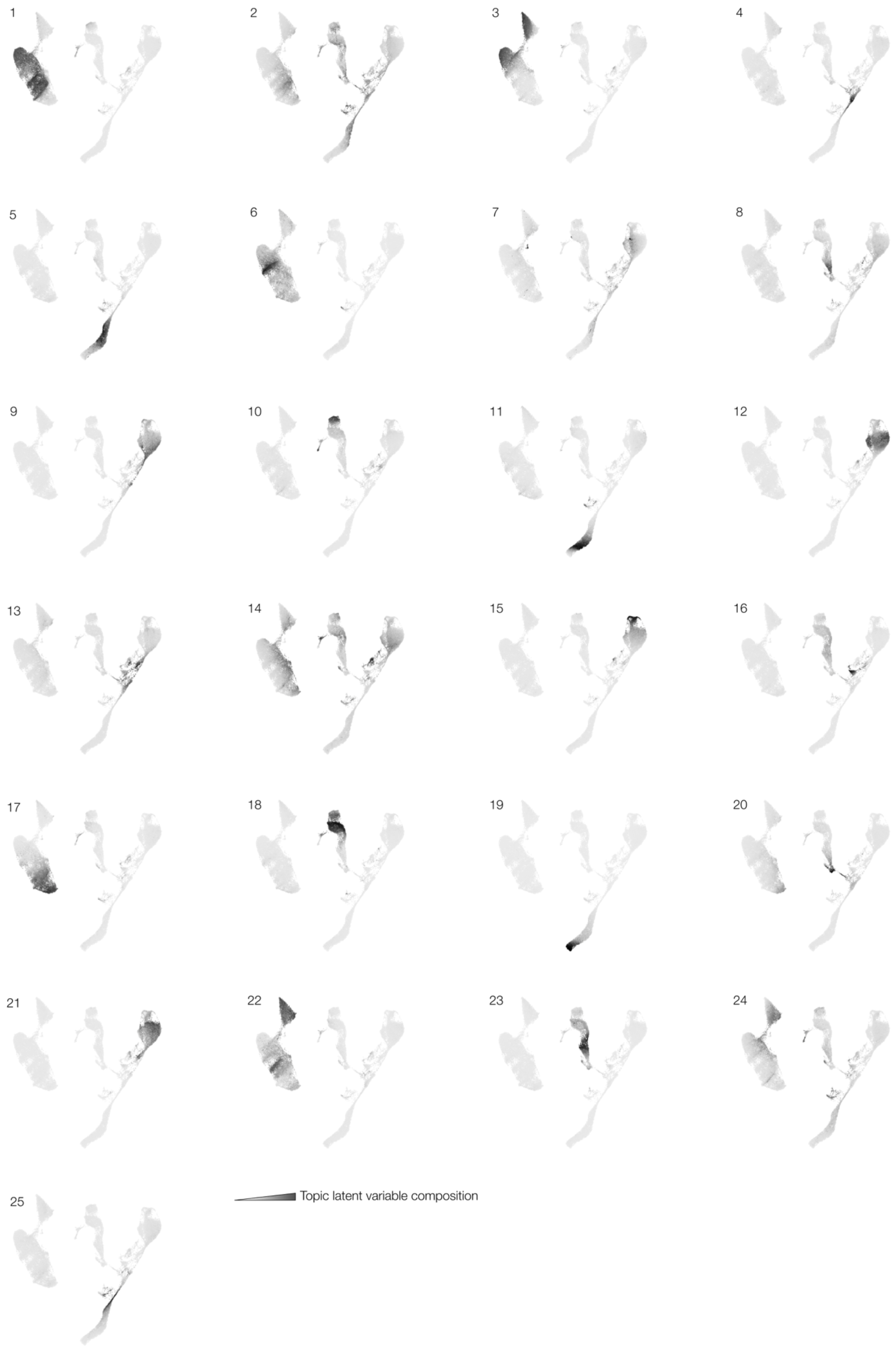

Supplementary Figure 7. CODAL latent topics from NEURIPS bone marrow ATAC

UMAPs colored by all (n=25) latent topics, *Z*, calculated from NEURIPS bone marrow ATAC-seq dataset using CODAL. Source data are provided as a Source Data file.

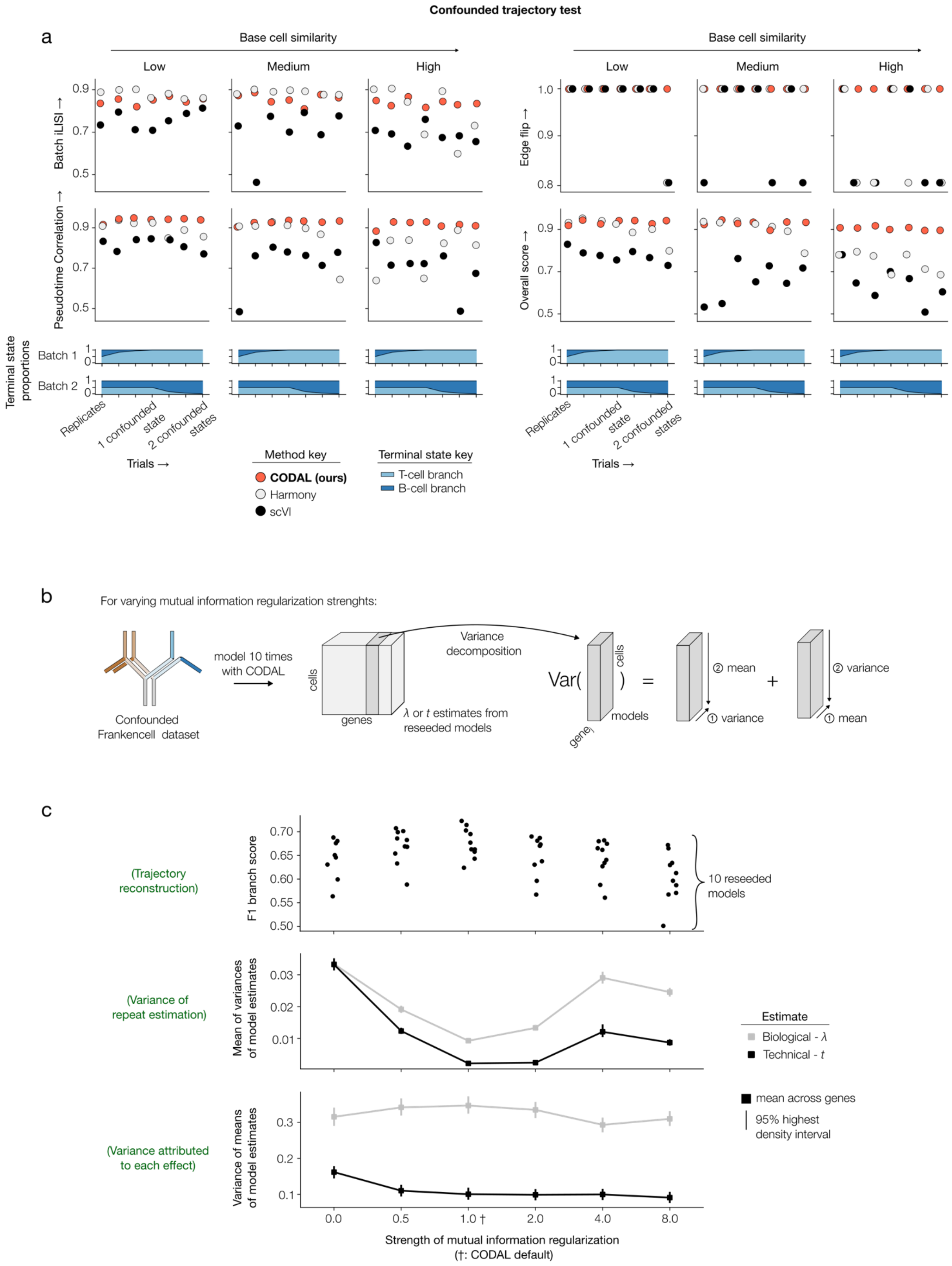

Supplementary Figure 8. Frankencell benchmarking metrics and model repeatability analysis

a) All other metric results for the “Frankencell” confounded trajectory test. (From top to bottom) Batch iLISI measures mixing of batches in the latent space and was only calculated for trajectory branches which were shared between both batches. Pseudotime correlation relates temporal geodesic distances between cells in the construction plan to the predicted trajectory. Finally, edge flip gives the edit distance in terms of edges added and removed to convert the predicted trajectory graph to the true graph, normalized so that a score of one means no edits were needed. With respect to each metric, better trajectory predictions earn greater scores. The “overall score” was calculated as the geometric mean of all metrics. Batch iLISI was calculated using the SCIB python package. All other metrics were calculated using the dynverse R package. **b)** Overview of variance decomposition analysis of biological and technical effect estimates from repeated CODAL modeling (with different initial seeds) of a batch-confounded Frankencell dataset. We varied the strength of the mutual information regularization term of the CODAL objective to investigate its effect on the repeatability and quality of the solution. When strength is zero, the CODAL objective reduces to marginal likelihood maximization; when strength is eight, the mutual information term is weighted eight times more highly than the default. The variance of biological or technical effect estimates for each gene are decomposed into the mean across cells of the variance of estimates, and the variance across cells of the mean of estimates. The first quantity measures how much the estimates of the model changed across multiple runs, the second measures, as an average of all models, the amount of variance ascribed to biological or technical effects across all cells. **c)** Results from variance decomposition analysis. Training models with the default CODAL objective reduced the variance of technical effect estimates across multiple runs 15-fold versus marginal likelihood maximization, while attributing 1.5-fold less variance to technical effects overall. Data are represented as the mean across n=1076 most highly variable genes (■). The error bar shows the 95% highest-density interval of the distribution across genes. The default CODAL objective also produced the most faithful trajectory reconstructions according to the F1 branch score metric. Source data are provided as a Source Data file.

a

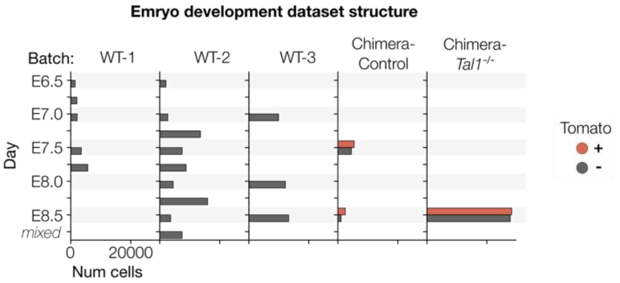
Emryo development dataset structure
Batch:
WT-1
WT-2
WT-3
Chimera-
Control
Chimera-
Tal1-/-
Day
E6.5
E7.0
E7.5
E8.0
E8.5
mixed
0
20000
Num cells
Tomato
+
-

b

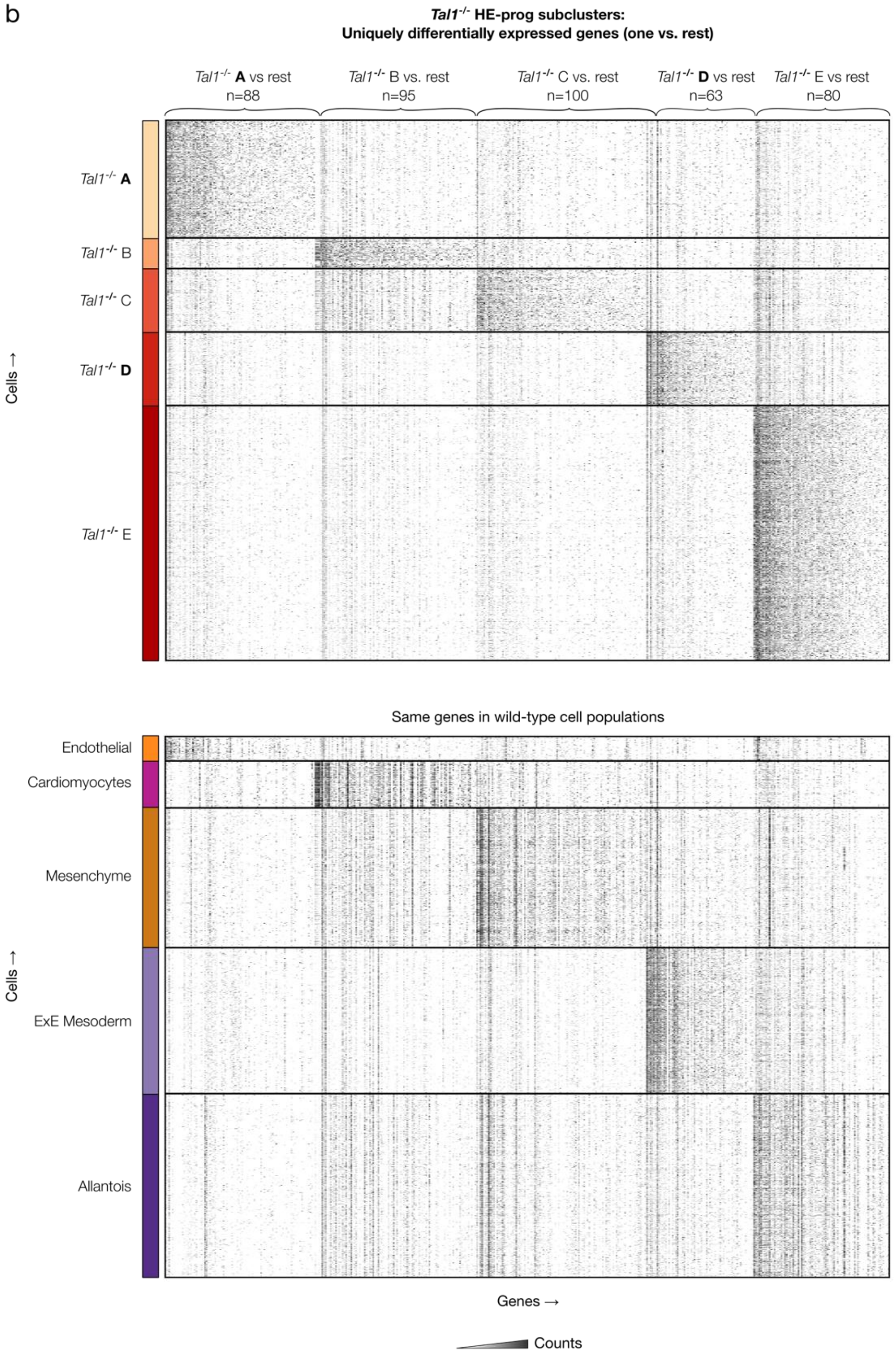
Tal1-/- HE-prog subclusters:
Uniquely differentially expressed genes (one vs. rest)
Tal1-/- A vs rest
n=88
Tal1-/- B vs. rest
n=95
Tal1-/- C vs. rest
n=100
Tal1-/- D vs rest
n=63
Tal1-/- E vs rest
n=80
Tal1-/- A
Tal1-/- B
Tal1-/- C
Tal1-/- D
Tal1-/- E
Cells →
Same genes in wild-type cell populations
Endothelial
Cardiomyocytes
Mesenchyme
ExE Mesoderm
Allantois
Cells →
Genes →
Counts

Supplementary Figure 9. Differential gene expression analysis of *Tal1*$^{-/-}$ haemato-endothelial progenitor subclusters

a) Number of cells sequenced from each day of development in each batch in the mouse embryo differentiation dataset, with bars colored by expression of Tomato. **b)** Heatmaps showing normalized expression counts for sets of differentially expressed genes called from each *Tal1*$^{-/-}$ subcluster. Differentially expressed genes were calculated using the two-sided Wilcoxon rank-sum test in a "one versus rest" manner, comparing the normalized expression of each gene in each of the *Tal1*$^{-/-}$ haemato-endothelial progenitor subclusters against expression in every other subcluster combined. For each subcluster A-E, we took the set of uniquely differentially expressed genes to be those genes which met the thresholds of $\log_2$ fold-change > 1.5 and p-value < 0.05 (Benjamini-Hochberg corrected), for only that cluster. (top) Expression of uniquely differentially expressed genes in cells from *Tal1*$^{-/-}$ subclusters. (bottom) Expression of same gene sets in cells from matched mesodermal cell type populations. Source data are provided as a Source Data file.

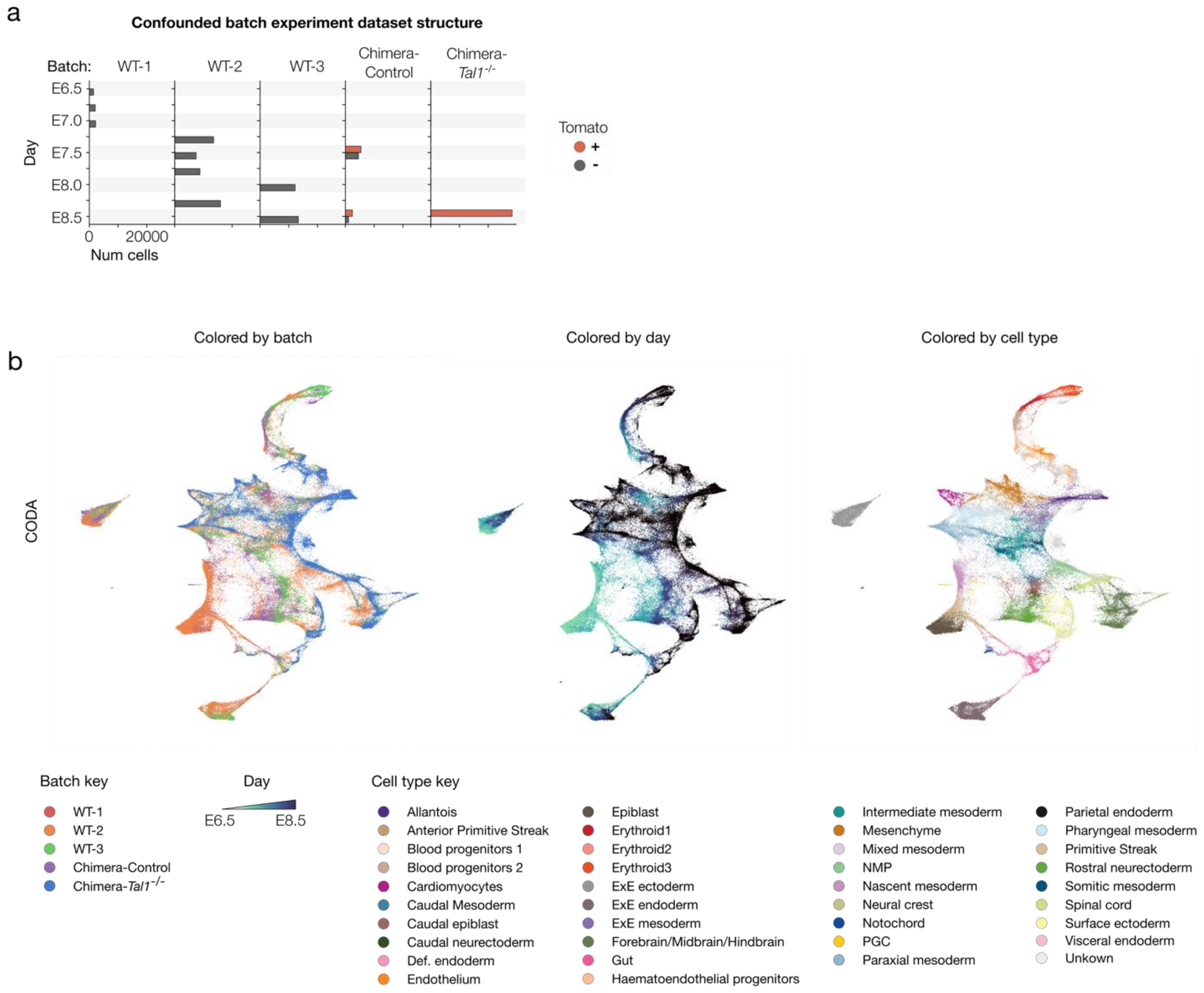

Supplementary Figure 10. UMAP representations of "confounded batch" down sampling of mouse embryo differentiation dataset

a) Number of cells sequenced from each day of development in each batch in the "confounded batch" experiment. Confounded batches were constructed by removing wild type Tomato$^-$ (*Tal*$^{+/+}$) cells from the chimeric mouse batch. In this way unperturbed haemato-endothelial progenitor and erythroid cell type populations, which overlapped with wild type cell types, were removed from the chimeric mouse. **b)** UMAP of CODAL latent space calculated from mouse embryo differentiation "confounded batch" dataset. From left to right, UMAP colored by cell batch of origin, day of differentiation at which cells were collected, and cell type annotations provided by the authors of the original study. Source data are provided as a Source Data file.
